# Crossing paths and avoiding catastrophe—an outsider’s inside view of yeast genetics

**DOI:** 10.1093/femsyr/foag009

**Published:** 2026-02-06

**Authors:** Daniel Hartl

**Affiliations:** Department of Organismic and Evolutionary Biology, Harvard University, Cambridge, MA, 02138, United States

**Keywords:** Hartl, Retrospective, yeast genetics

## Abstract

The substance of this article was presented at the 32^nd^ International Conference on Yeast Genetics and Molecular Biology. It traces the evolution of yeast genetics, its developmental milestones and influential figures. It highlights Daniel Hartl’s mentoring of and associations with postdoctoral fellows and key yeast investigators, from the hills of Berkeley to the halls of Harvard. It communicates Mario Polsinelli’s contributions to yeast population genetics and his role in rescuing a famous Tuscan winery from fermentation failure and financial desolation. It is a story of novel collaborative accomplishments, including DNA analysis of samples taken from ancient Egyptian wine jars and transcriptome analyses of vineyard yeast strains. It underscores the richness of yeast genetics and the immense impact of interdisciplinary curiosity and collaboration.

## Teaser

Retrospective by Daniel Hartl.

## I missed the first publication

It seems a bit awkward for me to be talking to an audience of professional yeast geneticists. This is because I came to yeast—or maybe I should say yeast came to me—somewhat late in my research career, and then with me as a mentor and collaborator rather than a hands-on bench researcher. This is by no means intended as a balanced history of yeast genetics of the sort one finds in Barnett (2007) or other papers. This is rather a personal reminiscence motivated by the comment usually attributed to George Sarton that “the history of a subject *is* the subject.”

**Figure 1:**
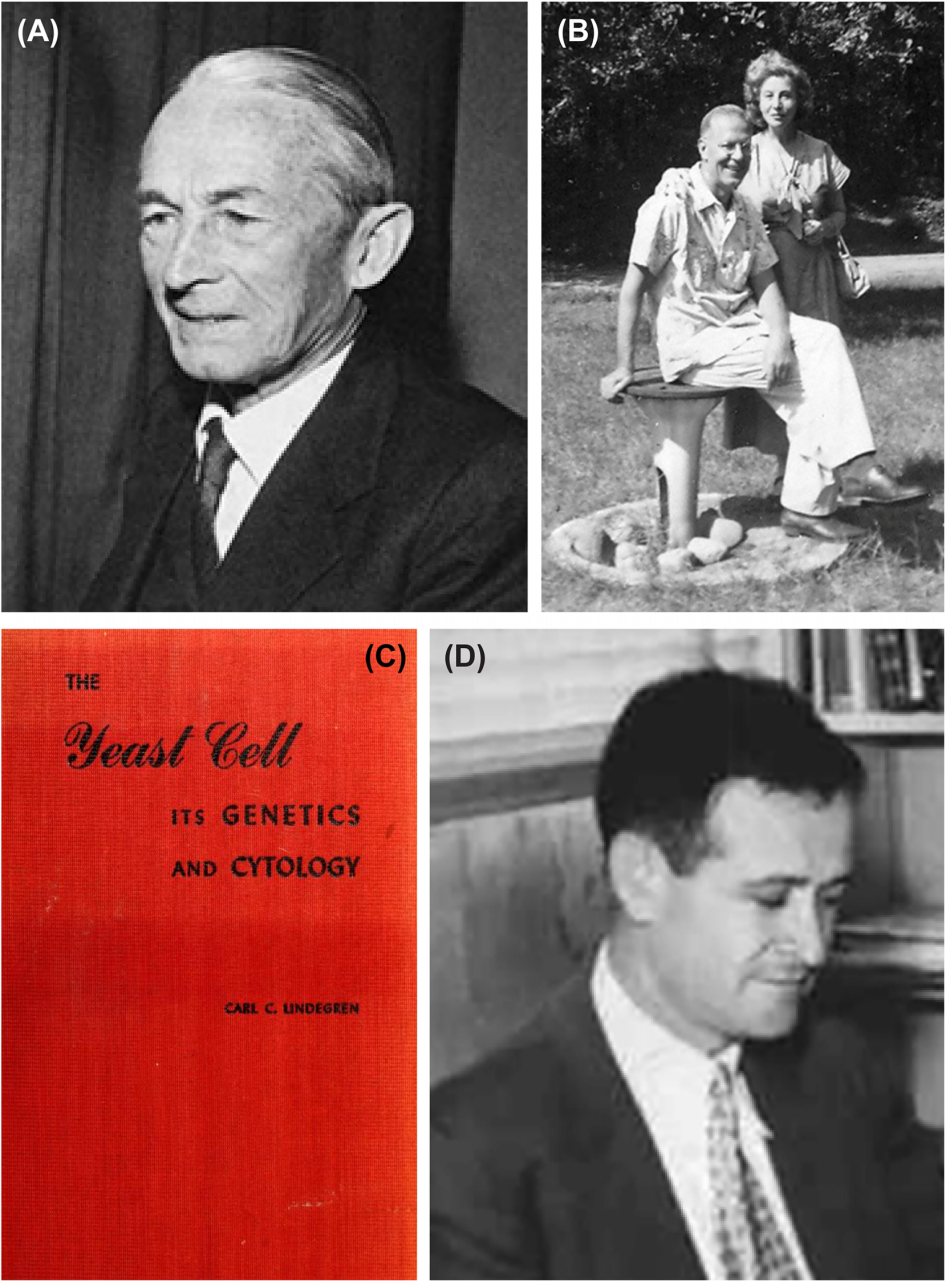
Investigators responsible for the birth of formal yeast genetics. **(*Panel A*)** Øjvind Winge (1886–1964). With permission of Wiley InterScience, J.A. Barnett (2007) A History of Research on Yeasts 10: Foundations of Yeast Genetics. Yeast 24:799–845. doi: 10.1002/yea.1513. There is a good photograph of Ojvind Winge at the Carlsberg Laboratory in Copenhagen in 1935. Køge Archives. https://koegearkiverne.dk/2127/oejvind-winge. **(*Panel B*)** Carl Lindegren (1896–1991) and his wife Gertrude, Rhinelander, Wisconsin 1949. With permission of Esther M. Zimmer Lederberg Memorial Website, *http://www.estherlederberg.com/EImages/Cold Spring Harbor/frames LindegrenCL CLindegrenGertrudeRhinelanderWis8-49.html*. **(*Panel C*)** T\Cover of the book Carl Lindegren authored containing the results of many of his early studies. Copyright Carl C. Lindegren 1949. Educational Publishers, Inc. St. Louis. **(*Panel D*)** Hershel Roman, who established yeast research at the University of Washington. With permission of Brian Giebel, University of Washington Genome Sciences. A later photograph of Hershel Roman may be found at Roman, H. (1986) The Early Days of Yeast Genetics: A Personal Narrative. Annual Review of Genetics. 20:1–14. doi: 10.1146/annurev.ge.20.120186.000245.

Although I am by no means a yeast geneticist, yeast genetics has been part of my professional life since as long as I can remember. As many of you know better than I do, modern studies of *Saccharomyces cerevisiae* began with the work of Øjvind Winge at the Carlsberg Laboratory in Copenhagen in 1935 (Fig. 1A) (Roman 1986). In the United States, pioneering research on budding yeast was begun by Carl C. Lindegren (1896–1991) and his wife Gertrude (Fig. 1B). The Lindegrens started their career working with *Neurospora* but then switched to yeast. Their first paper was published in 1943. As it happens, I was a baby in 1943, so I didn’t read the paper when it was first published.

## A first girl friend brought us together

Carl Lindegren grew up in Rhinelander, Wisconsin. His family was well known operating a very successful dry cleaning and laundry business called Lindy’s Laundry. Many of you may not know where Rhinelander is. I know the city well. It is in Northern Wisconsin about an hour’s drive north of Antigo where I grew up. My first serious girlfriend was from Rhinelander. When I was an undergraduate, I had a summer job at the United States Department of Agriculture’s Lake States Forest Experiment Station in Rhinelander, and while working there I stayed with my girlfriend’s family. Sadly, the Lake States Forest Experiment Station has since been reorganized and consolidated out of existence.

## Ghosting this guy

The Lindegrens carried out much of their research on yeast at Washington University in St. Louis from 1932 to 1954, after which they moved to Southern Illinois University Carbondale. Their early work is summarized in Lindegren’s 1949 book (Fig. 1C). Through a series of transpositions from one University to the next, I happened to be at Washington University in 1981, where Lindegren’s research had left a vivid legacy. As between Rhinelander and Washington University, I couldn’t help wondering why the science gods were making me ghost this guy.

## Lamarkism—no way

**Figure 2:**
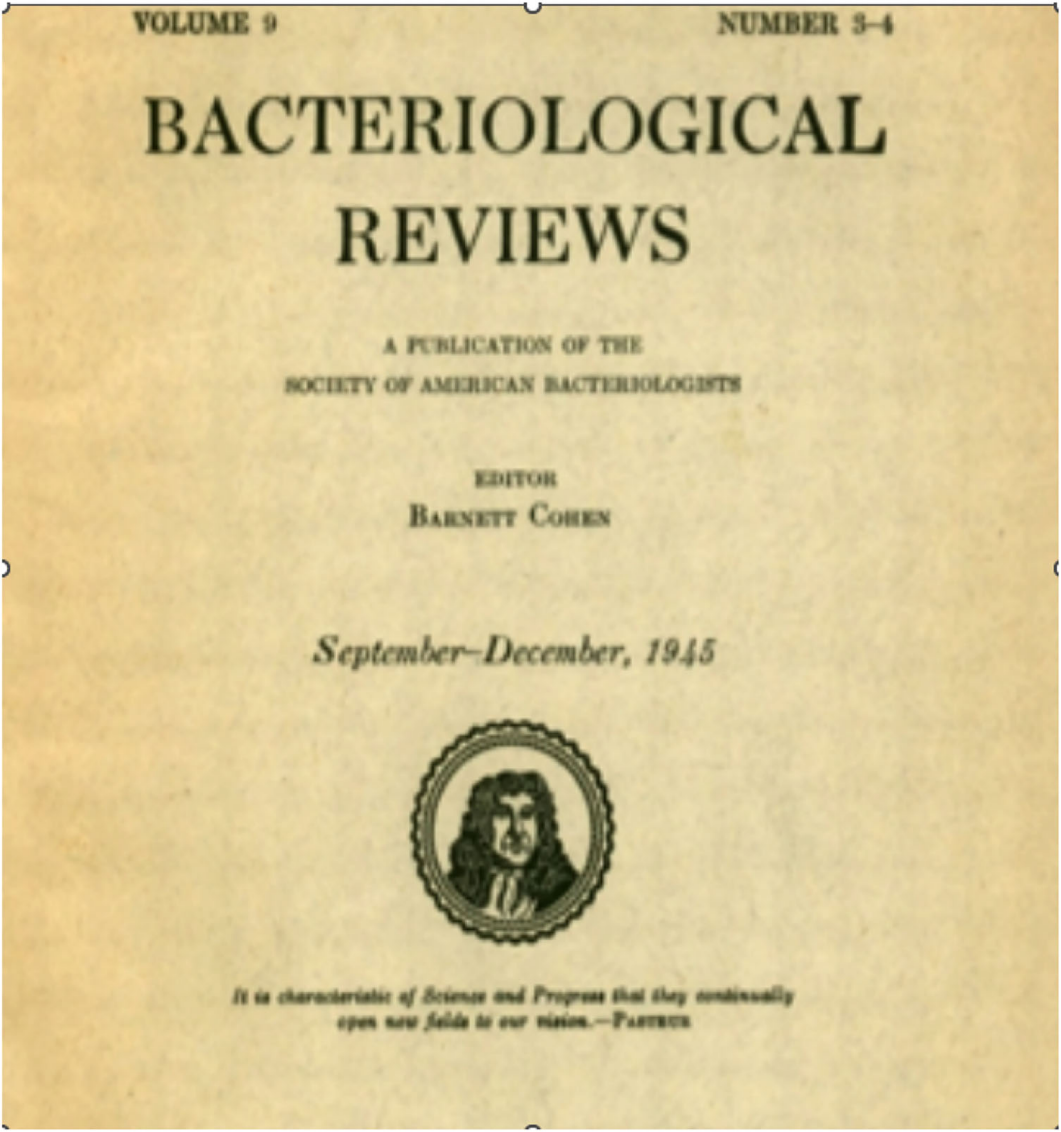
Cover of the 1945 Bacterial Reviews in which summarized much of Lindegren’s work.

By that time yeast research was already well established. The earliest focus of yeast research in the U.S. was in Herschel Roman’s group at the University of Washington in Seattle (Fig. 1D) (Stadler 1992). In 1947 he invited Lindegren to Seattle to offer his perspectives on yeast as an experimental organism. By that time Lindegren had recognized that yeast had two mating types, had developed methods for mating yeast strains and dissecting spores, had carried out genetic mapping experiments, and had discovered gene conversion. Much of this research is summarized in a 1945 paper in Bacteriological Reviews (Fig. 2) (Lindegren 1945), which cites many earlier papers dating back to 1943.

Lindegren’s work was nevertheless controversial because he strongly believed that yeast’s ability to adjust quickly to changes in environmental conditions was due to “adaptive mutation,” in which the cells direct their mutations as a response to environmental challenges. This idea smacked too much of Lamarkism. It was rejected straight away and didn’t raise its head again until John Cairns proposed adaptive mutation in *Escherichia coli* half a century later (1988).

## University of missouri—who would have thought

**Figure 3:**
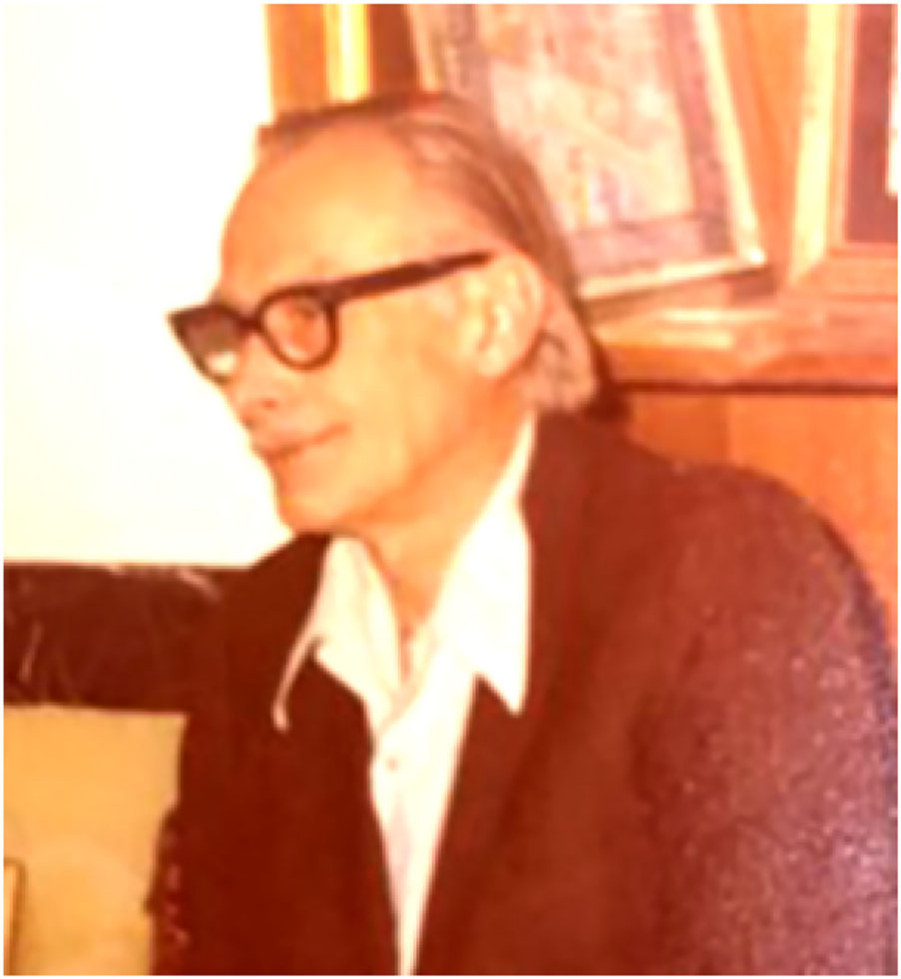
Spencer W. Brown.

Herschel Roman was a graduate student of Lewis J. Stadler at the University of Missouri in the early 1940’s (Roman 1986). His colleagues there included Barbara McClintock, Ernest R. Sears, George F. Sprague, Seymour Fogel, Irwin Herskowitz, Spencer Brown, and many other luminaries. And therein lies another of my outsider’s insider connection to yeast genetics. Spencer Brown was a student of Barbara McClintock, and in 1941 when she left Missouri for the Cold Spring Harbor Laboratory, she sent Spencer to the California Institute of Technology where he received his PhD (Fig. 3). After a few years on the faculty at U. C. Davis, Spencer moved to U. C. Berkeley where, in 1968–1969, I joined his group as a postdoctoral fellow. I well recall the many afternoons when we took our bag lunches out to the Berkeley hills Botanical Garden. Spencer seemed to know the common and scientific name of virtually every plant and talked at length about his experiences with McClintock and others at the University of Missouri. I didn’t get to know Barbara McClintock until many years later.

## The maestro and beginning of genetics in florence

**Figure 4:**
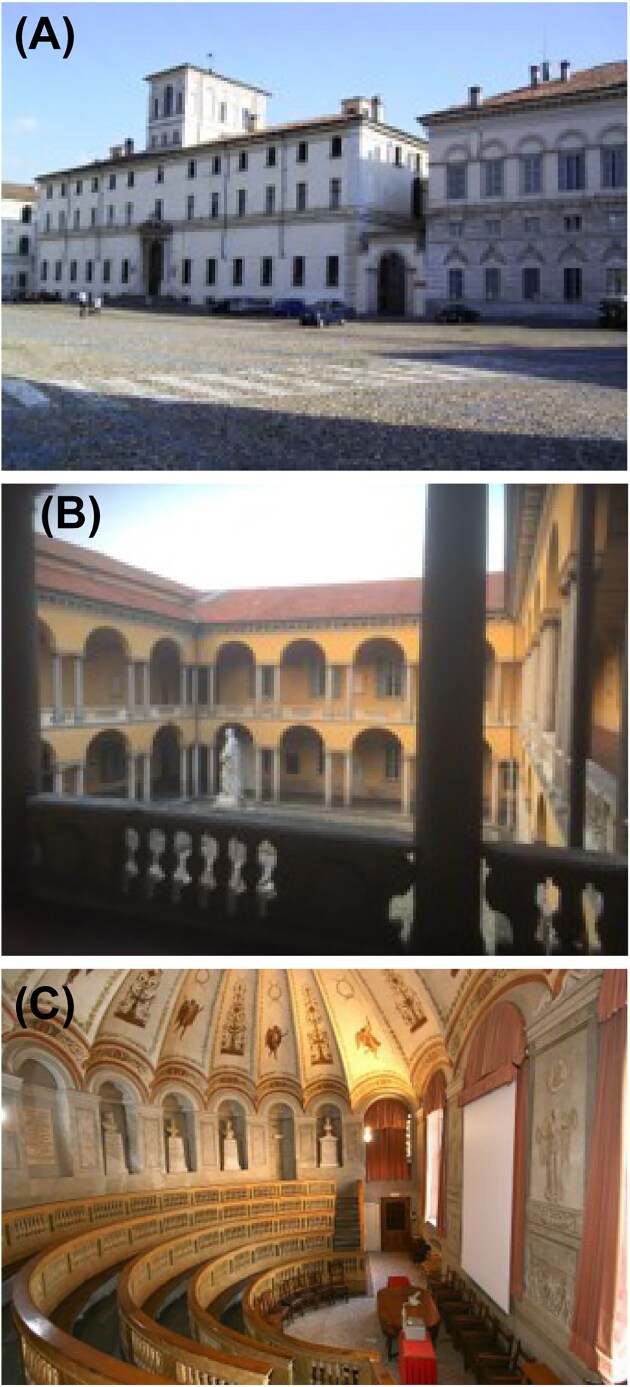
University of Pavia.

**Figure 5:**
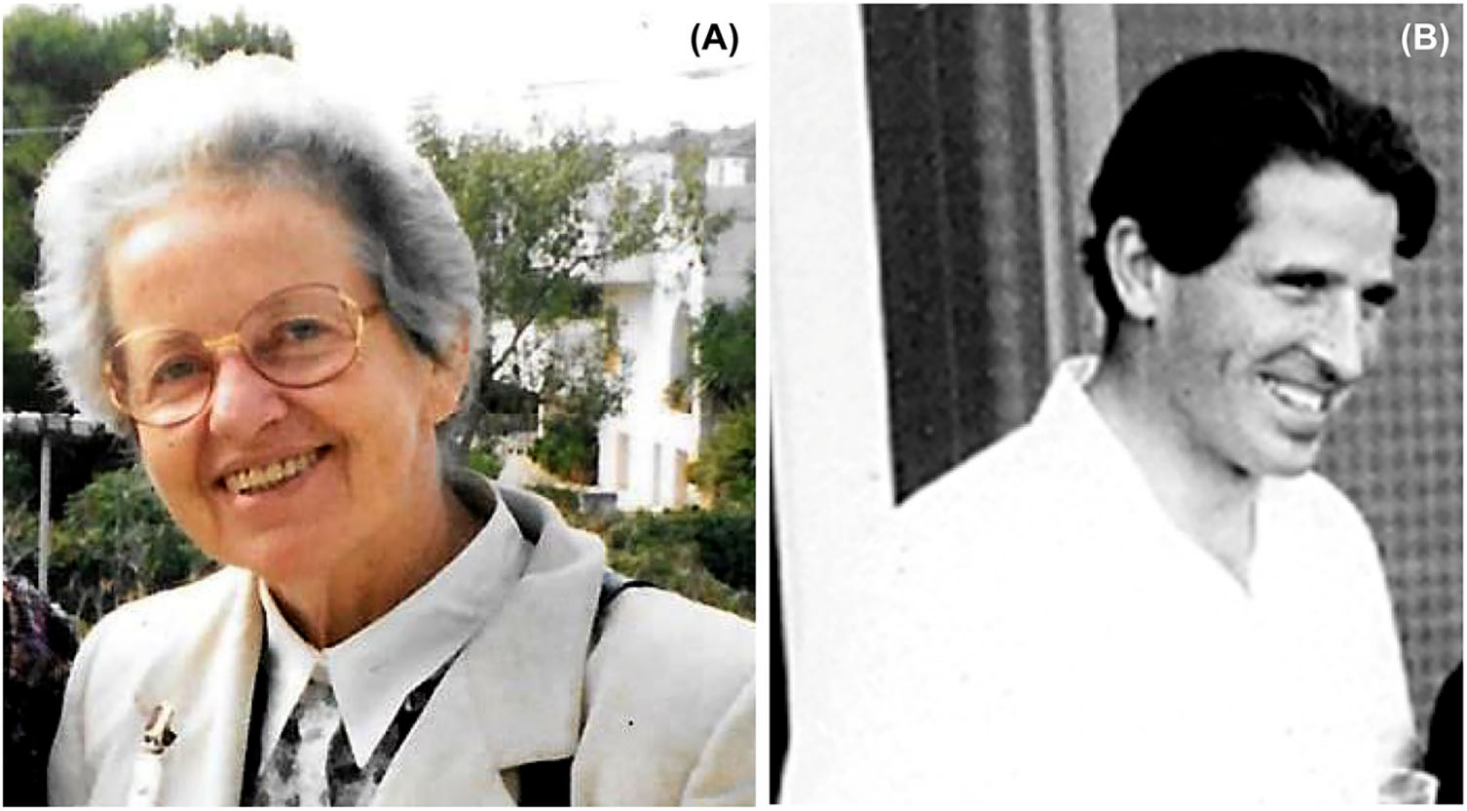
**(*Panel A*)** Laura Frontali. With permission of Oxford University Press; Laura Frontali, Laura Frontali—my life with yeast, *FEMS Yeast Research*, Volume 17, Issue 1, January 2017, fow107, https://doi.org/10.1093/femsyr/fow107 Fig. 3A. **(*Panel B*)** Mario Polsinelli.

While yeast genetics was becoming established in the U.S., an impressive group of young scholars had assembled in the Institute of Genetics at the University of Pavia in Italy (Fig. 4). The group included Adriano Buzzati-Traverso, Luca Cavalli Sforza, Giovanni Magni, and Mario Polsinelli. All established distinguished careers elsewhere: Cavalli-Sforza at Stanford, Buzzati-Traverso at Naples (see Frontali 2017, for her remembrances; Fig. 5A), Magni at Milan, and Polsinelli at Florence.

It’s Mario Polsinelli who I’d like to emphasize here (Fig. 5B). He was born in 1924 in Arpino, a small village about 75 miles southwest of Rome. He studied agriculture in Naples and then from 1948–1963 carried out research on the nearly 600 varieties of Italian wine grapes. In 1963 he joined the other luminaries at Pavia. When Cavalli-Sforza left Pavia, Polsinelli took a sabbatical year to join him and Joshua Lederberg at Stanford. On his return, he became director of the Institute of Genetics, where he remained until 1974 when be moved to Florence and founded the Department of Genetics as well as the Florentine Microbial School of Genetics at Cortona in Tuscany.

I emphasize Polsinelli for two reasons. First, because he will enter this story again a little later. And second, unlike yeast geneticists in the U.S., whose main emphasis was on laboratory strains and their mutants, Polsinelli focused on naturally occurring yeast lineages. Through the years he accumulated a vast collection of naturally occurring *S. cerevisiae* from numerous vineyards and other sources. He became the maestro of natural yeast isolates, which has had important practical and economic consequences for vintners.

## Setting up the holliday model

**Figure 6:**
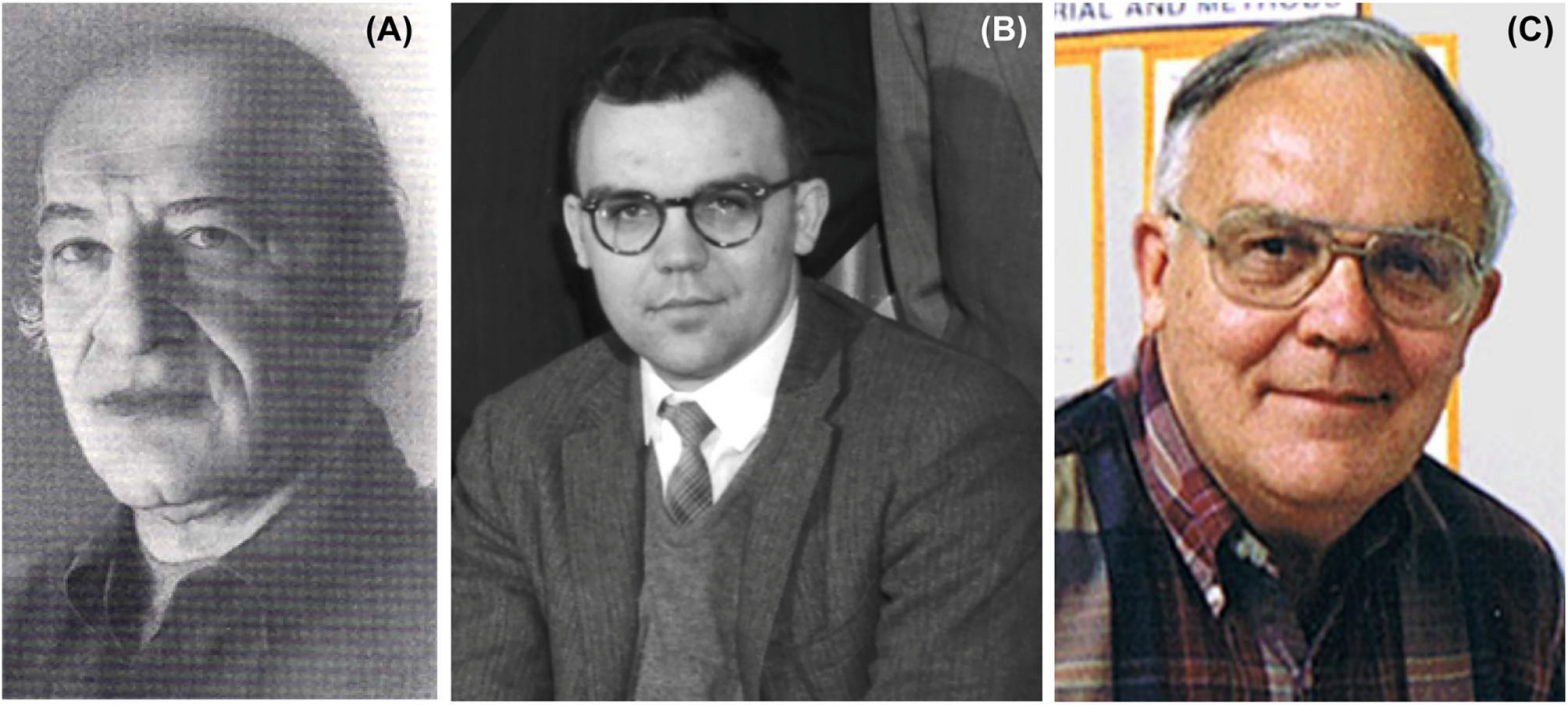
**(*Panel A*)** Seymour (Sy) Fogel. With permission of John Wiley and Sons, Ltd., M. S. Esposito, T. G. Cooper, P.P. Slonimski (1994) Obituary. In memory of Seymour Fogel. Yeast 10(7):975–977. PMID: 7 985 425 DOI: 10.1002/yea.320100713. **(*Panels B)*** Robert Bob Mortimer taken January 11, 1960. Open Access. Wikipedia. Photograph by Donald Cooksey, Donner Laboratory. Morgue 1960–63 Pg1. Lawrence Berkeley National Laboratory, https://en.wikipedia.org/wiki/Robert_K._Mortimer#/media/File:Robert_K._Mortimer,_1960.jpg. ***( Paneld C*)** Robert C. (Bob) Mortimer. With permission of Robert Sanders, UC Berkeley, Media Relations, https://mcb.berkeley.edu/news-and-events/department-news/robert-mortimer.

I’ll now return to 1968–1969 and my postdoctoral year at Berkeley. At that time U.C. Berkeley was split into many small departments. I attended group meetings in genetics, where I was with Spencer Brown; and zoology, where I got to know Curt Stern and Eva Sherwood; and the yeast group headed by Seymour Fogel who had just moved from Brooklyn College to become Chair of Molecular and Cell Biology (Fig. 6A). Sy had become a convert from maize genetics when in 1958 he spent a sabbatical in Seattle with Herschel Roman learning yeast genetics. He spent the following years carrying out early studies of gene conversion that set the stage for Robin Holliday’s 1964 model of recombination. Sy then spent the following years documenting further aspects of gene conversion, highlighting its fidelity, parity, polarity, and relation to recombination while providing evidence against the Holliday model in its simplest form and contributed a treasure trove of tetrad analyses data to other investigators that ultimately led to today’s double-strand break-and-repair model.

## The most well-known physicist in yeast genetics

Also in the yeast group was Robert K. Mortimer (Fig. 6B, C). Bob was trained as a physicist at the University of Alberta in Canada. In his early research at Berkeley, he explored the mechanisms used by yeast to repair radiation and chemical damage to its chromosomes and discovered the now-famous RAD genes. He also pioneered techniques that made yeast easier to study, such as the use of juice from snail guts to digest the tough outer shell of the ascus, and he established a large collection of yeast strains and mutants that he freely shared across the entire yeast community.

Mortimer had always been interested in yeast natural history, and upon his retirement in 1991, he turned his attention to the strains of wine yeast. He thereupon became affiliated with the University of Florence and spent much of his time in the vineyards of Tuscany and other European centers of winemaking.

## Duccio you really don’t want that

**Figure 7:**
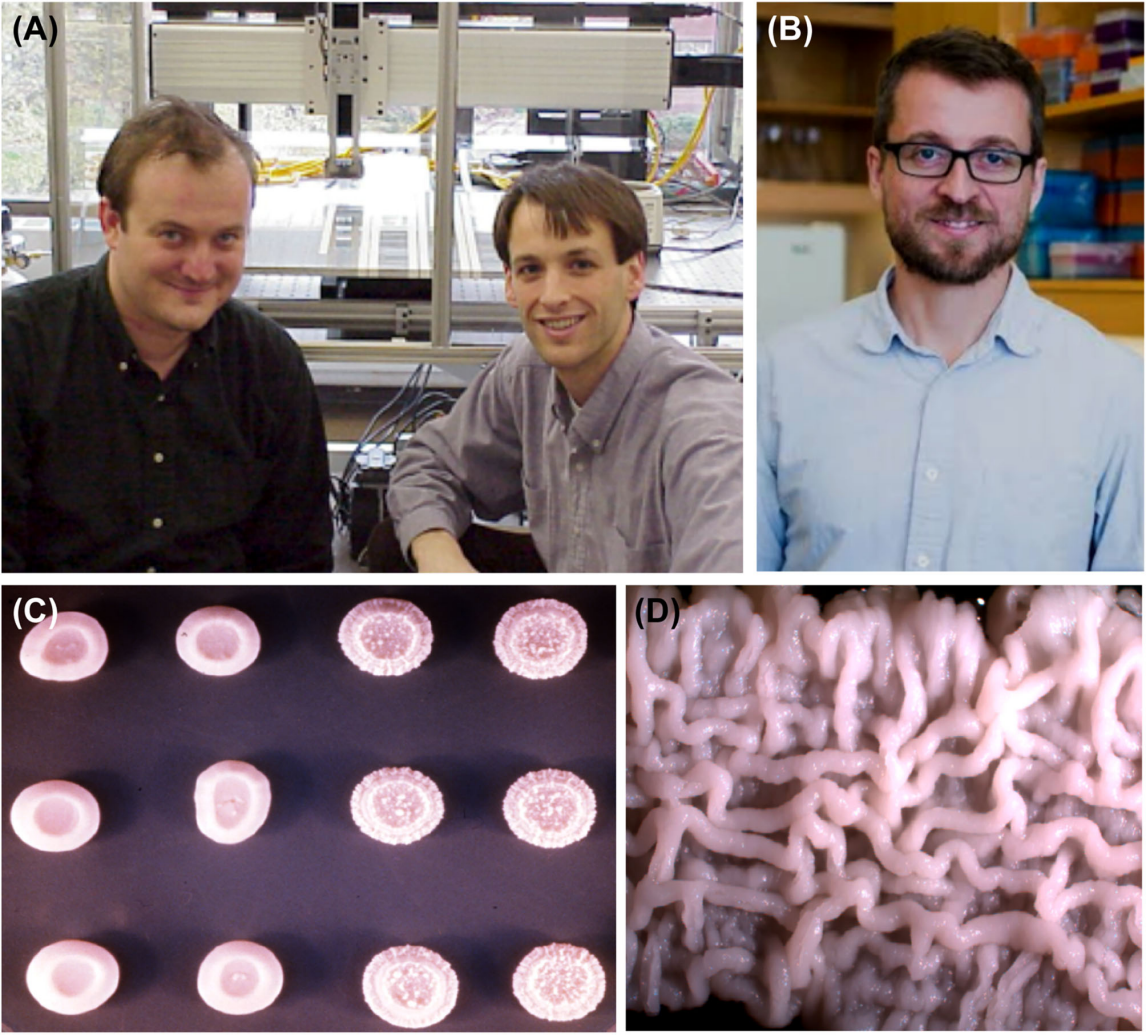
**(*Panel A*)** Duccio Cavalieri and Jefferey Townsend in the laboratory of Daniel Hartl. **(*Panel B*)** Christian (Chris) Landry. **(*Panels C and D*)** a strain isolated from wine grapes in a Tuscan vineyard that was heterozygous for a gene that segregated colonies with a ridged surface resembling a filigree.

Bob Mortimer also did me one of the great scientific favors of my life when in 1999 he talked Duccio Cavalieri out of taking a faculty position and instead encouraged him to join my research group at Harvard (Fig. 7A). Duccio did indeed join my group at Harvard, where for three delightful years he and several of my students, including Jeffrey Townsend (Fig. 7A) and Christian Landry (Fig. 6B), were highly productive in studying natural isolates of *S. cerevisiae*. The studies included comparative gene expression and a refined statistical analysis for comparing gene expression levels in natural isolates. They also included studies of a strain isolated from wine grapes in a Tuscan vineyard that was heterozygous for a gene that segregated colonies with a ridged surface resembling a filigree (Fig. 7C, D). Global expression analysis of the progeny with the filigreed and smooth colony phenotypes revealed a >2-fold in transcription for 378 genes (6% of the genome). A large number of the overexpressed genes function in pathways of amino acid biosynthesis (particularly methionine) and sulfur or nitrogen assimilation, whereas many of the under expressed genes encode amino acid permeases (Cavalieri et al. 2000).

## Best drug around

**Figure 8:**
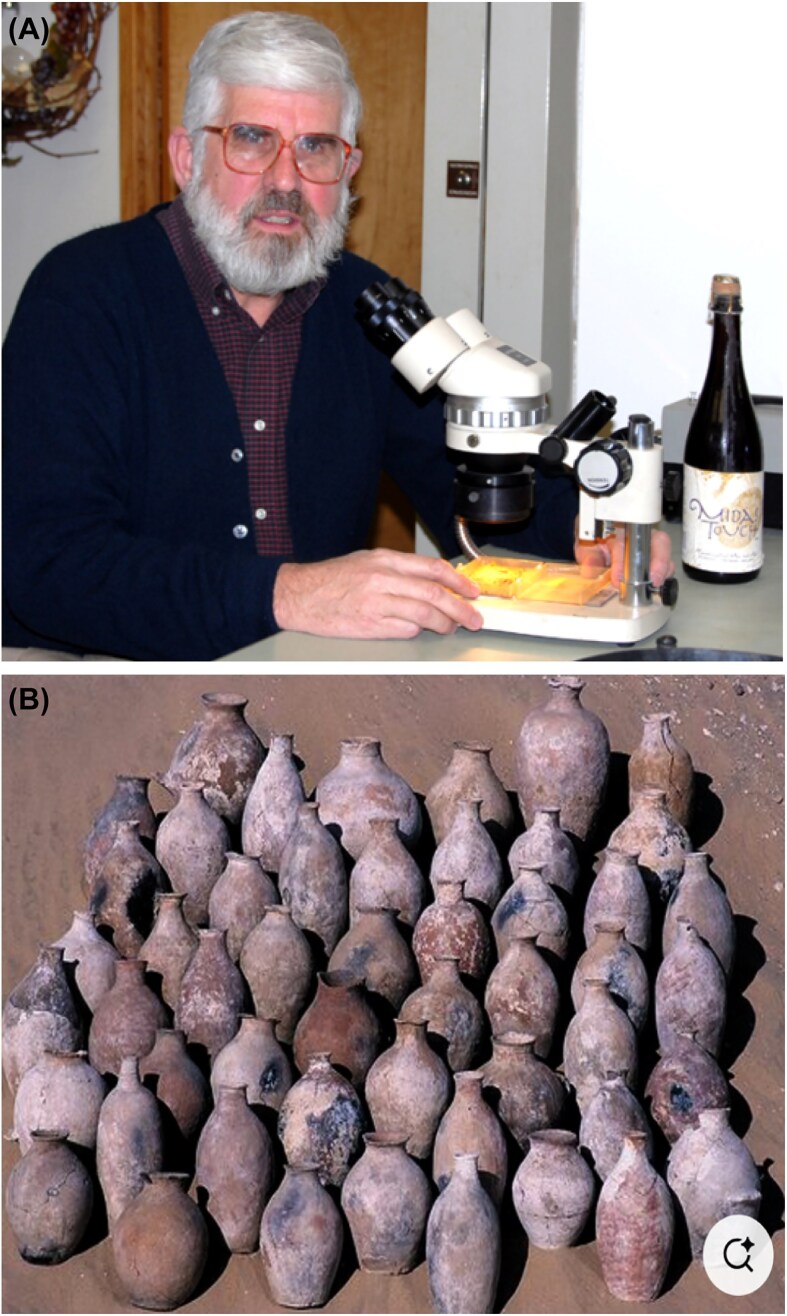
**(*Panel A*)** Patrick McGovern, the Indiana Jones of ancient fermentation. **(*Panel B*)** Wine jars from the tomb of Egyptian King Scorpion I who reigned 3300 BC.

After his initial stay, Duccio returned to Harvard at frequent intervals, often bringing new projects or new collaborations along. One of the most memorable to me was a collaboration with the archaeologist Patrick E. McGovern (Fig. 8A), an expert on ancient ales, wines, and extreme beverages. Winemaking, as he has pointed out, produced the most widespread drug and medicine of antiquity owing to its high content of ethyl alcohol and its accompanying mind-altering, analgesic, disinfectant, and preservative properties.

## 5 000-year-old *S. cerevisiae*

McGovern had gained access to residue from one of the hundreds of wine jars deposited in the tomb of Scorpion I, one of the first kings of Egypt, at the site of Abydos along the middle Nile River. The wine jars dated to ∼5300 years ago and had been imported from the southern hill country of Palestine and the Jordan Valley (Fig. 8B). If all the jars were full when imported, as seems likely, they originally contained the equivalent of 6000 of today’s wine bottles. The mouths of the jars had been sealed and were well protected from the sun in the extremely low-humidity environment of the Western Saharan Desert of Upper Egypt, which helped to preserve the fragments of DNA and other organic material in the jars, including dried figs and raisins.

Working in a clean room free from microbial contamination, Duccio had successfully amplified an 840-bp sequence of DNA consisting of 394 bp of the ITS1 (internal transcribed spacer 1), the 158-bp sequence encoding 5.8S RNA (RDN58-1), and 288 bp of the ITS2. The yeast genome contains 100–200 copies of this sequence, embedded in a tandemly repeated unit of 9.1 kb that encodes the 18S, 5.8S, and 25S rRNAs. The fragment was gel purified and sequenced and found to be unequivocally *S. cerevisiae* to the exclusion of other yeast species as well as being significantly different from the corresponding sequence in extant isolates. The conclusion is that ancient fermentation relied on *S. cerevisiae* as fermentation still does today (Cavalieri et al. 2003).

## Avoiding a million dollar catastrophe

**Figure 9:**
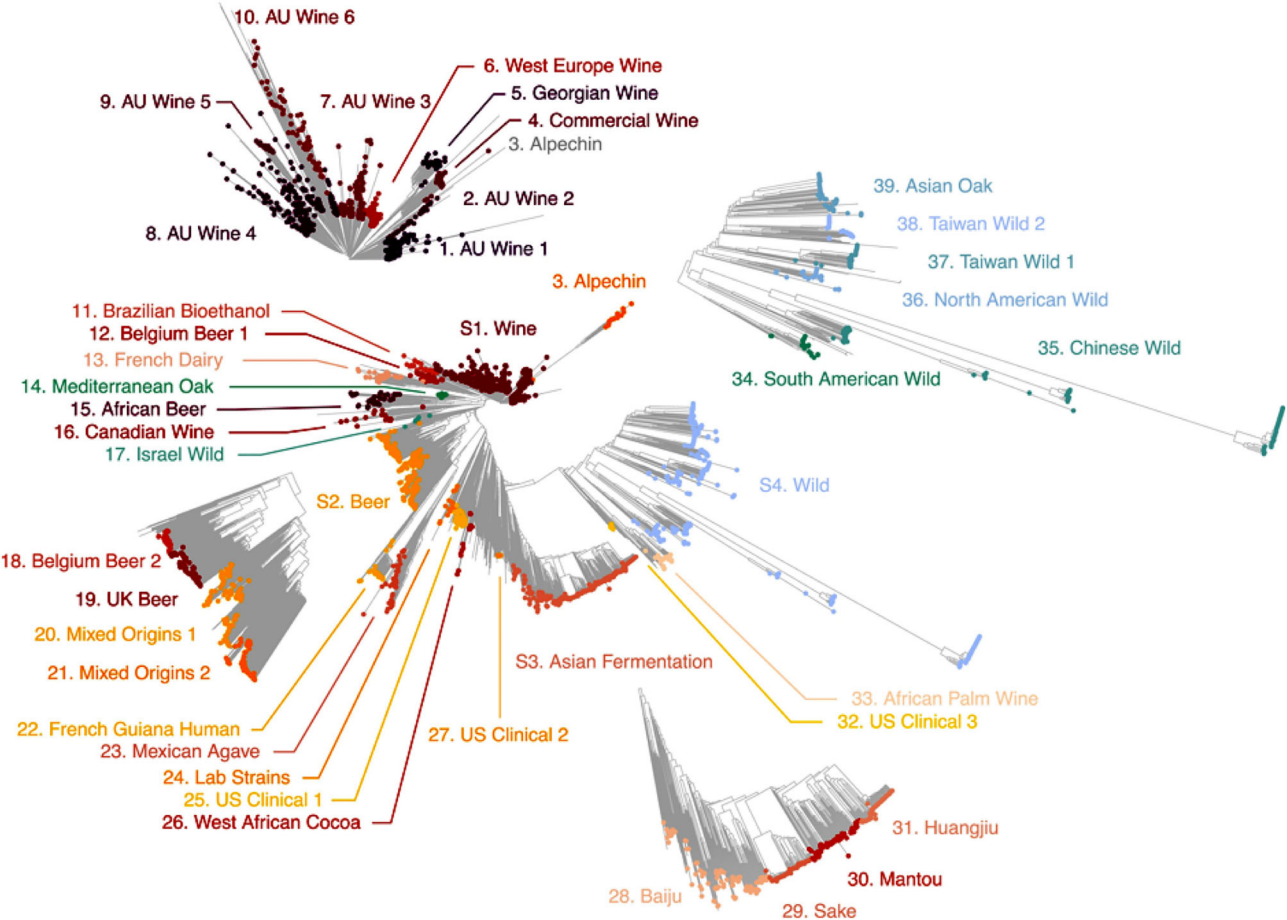
Overview of the *S. cerevisiae* population structure through the lens of 3034 genomes. Oxford University Press, Open Access, V. Loegler, A. Friedrich, J. Schacherer (2024) G3 Genes|Genomes|Genetics, 14: jkae245, https://doi.org/10.1093/g3journal/jkae245.

**Figure 10:**
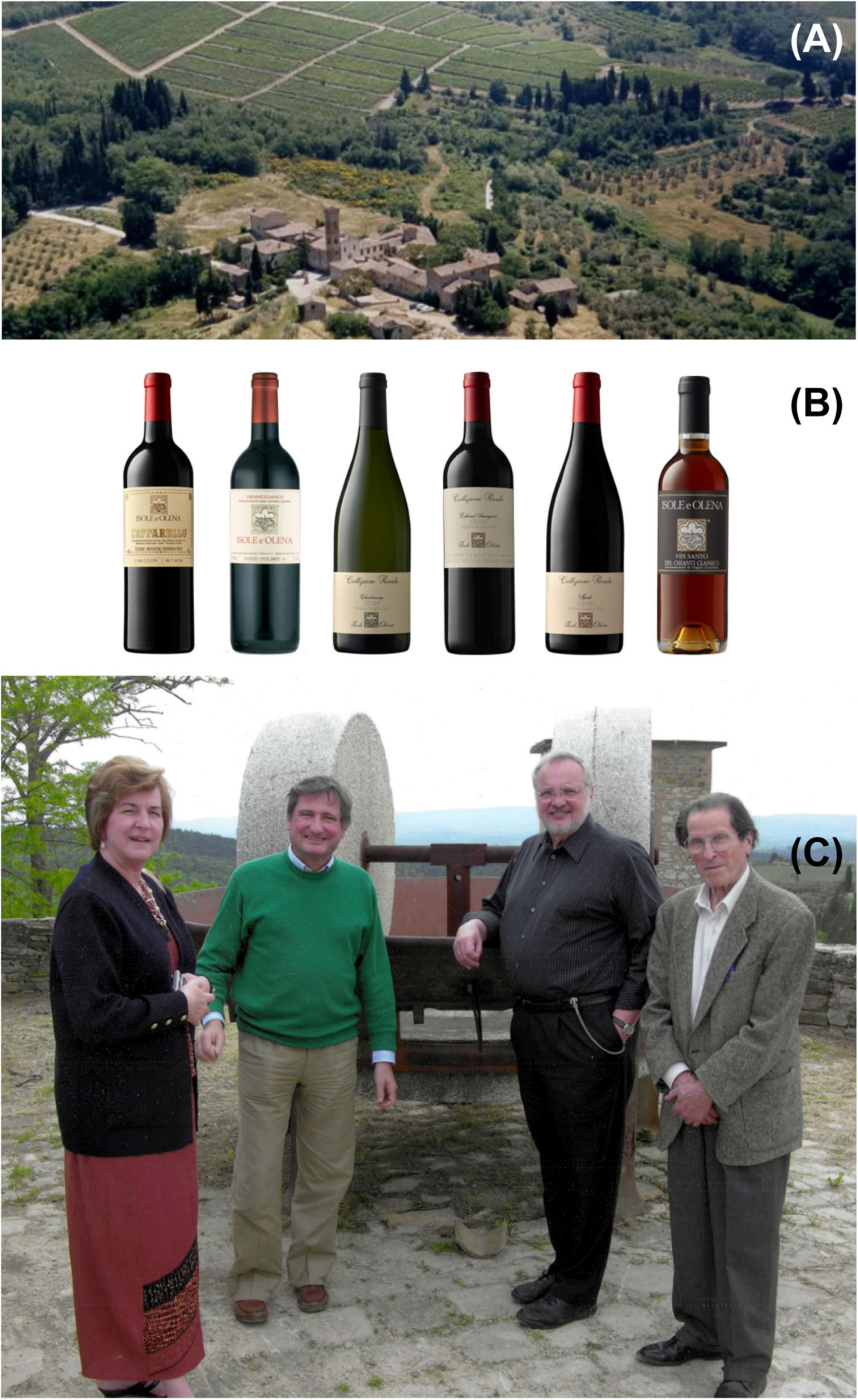
**(*Panel A*)** Isole e Olena winery. **(*Panel B*)** Isole e Olena wines. **(*Panel C*)** Left to right, Christine Blazynski, Paolo de Marchi, Daniel Hartl, and Mario Polsinelli.

All this while, I was unable to travel to Florence until May of 2009. There I finally met Mario Polsinelli, the maestro, the real founder of yeast population genetics. He had established a vast collection of naturally occurring yeast strains (Fig. 9). The importance of these strains is exemplified by his role in averting an impending catastrophe at what was arguably the premier winery in Tuscany. The winery is Isole e Olena and its proprietor was Paolo de Marchi (Fig. 10A). Isole e Olena produced some of the finest wines in the world, including a cold-fermented dessert wine known as Vin Santo (Fig. 10B). Paolo invited Mario, Duccio, my wife and I out to the winery (Fig. 10C). We had a lovely get-together and lunch and then went down to business. Paolo had three gigantic stainless-steel tanks filled with grape must intended to cold ferment into Vin Santo. The problem was that no fermentation was taking place. Could Mario possibly help?

Just before we left the winery, Mario collected several huge jugs of the must and took them along. On returning to his lab in Florence, he set out to find yeast strains that would act as fermentation starters. Guided by experience, time and place of collection, and instinct, he chose a few hundred candidate strains and tested them individually in fermentation flasks at low temperature. He found several strains that would ferment the must, and he created a mixed slurry to try at the winery. And it worked. The catastrophe was averted. How much was his effort worth? Well, a bottle of Isole e Olena Vin Santo sells for about $80 per bottle, so one could safely say that his efforts were literally worth millions of dollars.

I might add here that Paolo de Marchi had to sell the property of Isole e Olena a few years later. He’s currently putting his expertise to good use helping his son Luca De Marchi craft gorgeous, distinctive wines at his family’s estate at Proprietà Sperino in Lessona near Milan.

## Peeking through the keyhole is good

**Figure 11:**
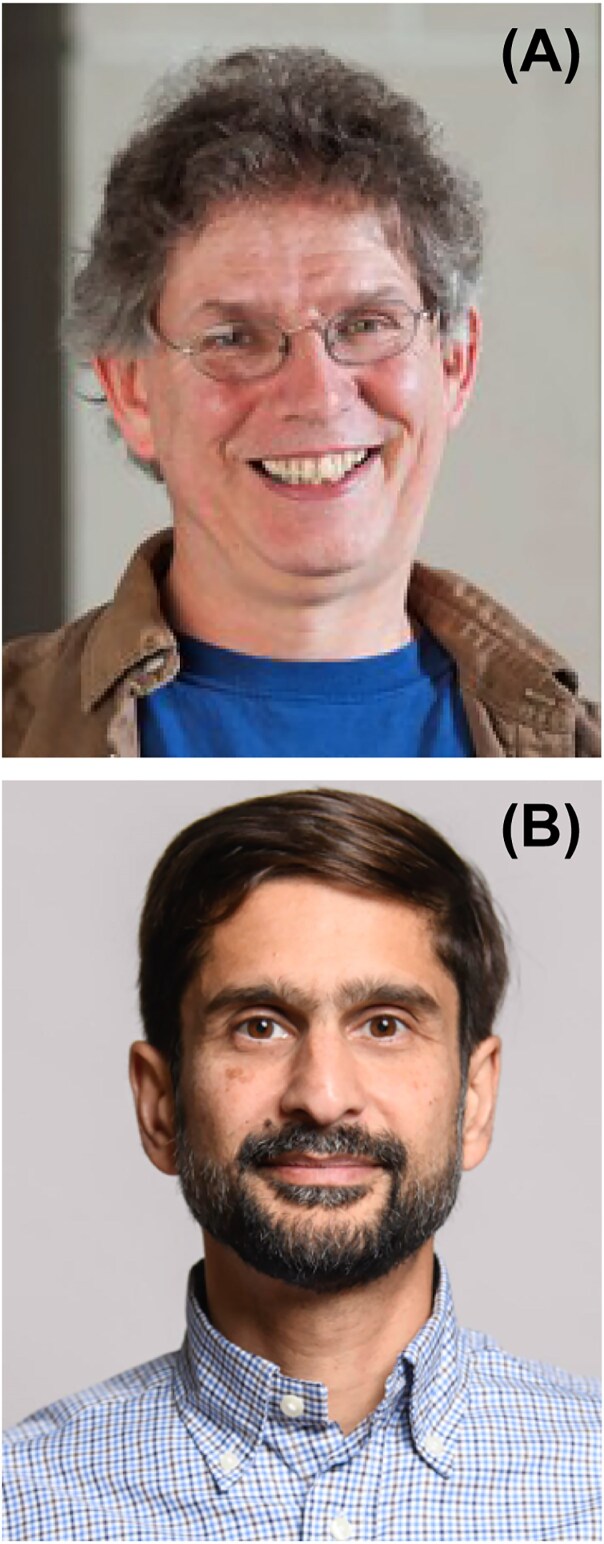
Colleagues of Daniel Hartl at Harvard University. **(*Panel A*)** Andrew Murray. **(*Panel B*)** Michael Desai.

I’d end here, but it’s not quite the end. As I’ve made clear throughout this chronicle, as an outsider I’ve been privileged to be able to witness the growth and success of yeast genetics and molecular biology and population biology by peeking through the keyhole, as it were. And my good luck has continued, because in my present position I am privileged to have as my colleagues two of the world’s finest yeast researchers, namely Andrew Murray and Michael Desai (Fig. 11A and B).
